# Molecular analysis of small-ruminant lentiviruses in Polish flocks reveals the existence of a novel subtype in sheep

**DOI:** 10.1007/s00705-019-04161-9

**Published:** 2019-02-09

**Authors:** Monika Olech, Maciej Murawski, Jacek Kuźmak

**Affiliations:** 1 Department of Biochemistry, National Veterinary Research Institute, Al. Partyzantów 57, 24-100 Puławy, Poland; 2 Department of Animal Biotechnology, Agricultural University of Kraków, 1B Rędzina, 30-248 Cracow, Poland

## Abstract

Small-ruminant lentivirus (SRLV) infections are widespread in Poland, and circulation of subtypes A1, A12, A13, A16, A17, B1 and B2 has been documented. The aim of this study was to characterize the SRLV strains circulating in sheep and goats in mixed flocks in the Malopolska region, where the highest seroprevalence has been detected. Phylogenetic analysis revealed that most of the isolates from sheep belonged to subtype A13, suggesting that this subtype may be predominant in the Malopolska region. Furthermore, the existence of a new subtype, tentatively designated as A18, was described for the first time. This work extends the current knowledge on the distribution of SRLV subtypes in sheep and goats in Poland and provides further information on the genetic diversity of SRLV. The new data are important for both epidemiological studies and eradication programs and provide insight into the evolution of SRLV.

Small-ruminant lentiviruses (SRLV), whose prototypes are caprine arthritis encephalitis virus (CAEV) and maedi visna virus (MVV), belong to the genus *Lentivirus* of the family *Retroviridae*. These viruses, which naturally infect goats and sheep, cause a persistent infection that induces chronic inflammatory and degenerative disease of the lungs, joints, central nervous system, and mammary glands [1–3]. SRLV infections have an impact on the sheep and goat industry, causing considerable economic losses related to reduced milk production and weight gain as well as increased mortality in the offspring before weaning [4, 5].

The genome of SRLV, which is integrated into host cells in the form of a provirus, contains three structural genes, *gag* (group-specific antigen), *pol* (polymerase) and *env* (envelope), as well as the accessory genes *tat*, *vif* and *rev*, which have regulatory functions. Phylogenetic analysis based on a 1.8-kb sequence of the *gag-pol* region and a 1.2-kb *pol* gene sequence divides these viruses into five major groups, A-E. MVV-like and CAEV-like viruses, classified in groups A and B, respectively, are widely distributed throughout the world, while viruses from groups C-E are geographically restricted [6–8]. Seventeen subtypes (A1–A17) have been recognized within group A so far, whereas group B is divided into four subtypes, B1–B4 [9–11]. Studies conducted in 2012 and 2018 showed that Polish strains belong to the well-known subtypes B1, B2 and A1 as well as to the more recently established subtypes A12, A13, A16 and A17 [11, 12].

SRLV are characterized by a high degree of genetic variability, leading to the occurrence of a variety of divergent strains and quasispecies [10]. Moreover, an additional mechanism contributing to the growing diversity of SRLV is the ability to infect both sheep and goats. Multiple studies have described the phenomenon of cross-species transmission, which may drive the emergence of new strains, possibly displaying new biological properties [13, 14]. Detailed knowledge about the genetic diversity of SRLV and the genotypes circulating in the field is crucial for the effectiveness of control programs as well as for the development of diagnostic tests for detection of local strains [15].

Previous serological surveys showed that SRLV infections are widespread in Poland and that the prevalence at the flock level reached 71.9% and 33.3% in goats and sheep, respectively [16, 17]. The latter study also documented differences in the proportion of infected animals between geographical regions, with the highest seroprevalence of 72% in sheep in the Malopolska region. This region, located in the south of Poland, has the highest population of sheep, accounting for close to 30% of the total population, and traditional breeding of sheep and goats in mixed flocks, where both species are housed together is common. Since the housing of animals in mixed flocks promotes interspecies transmission [18, 19] and can lead to the emergence of new genetic variants, the aim of this study was to carry out a molecular analysis of SRLV strains in mixed flocks of sheep and goats in the Malopolska region.

Blood samples were collected from 68 sheep and 14 goats, selected from four mixed flocks where infection with SRLV was recognized based on previous serological testing by ELISA [12]. This mixed flocks represents 3.3% of the total number of flocks located in the Malopolska region. All animals were clinically healthy and came from closely located flocks. Peripheral blood leukocytes (PBL) were isolated from 10 ml of blood by centrifugation at 1500 *g* for 25 min. The buffy coat was collected and subjected to osmotic hemolysis using cold water and 4.5% NaCl. After two washes in PBS, the supernatant was discarded and the cell pellet (5×10^6^ cells) was used for extraction of genomic DNA using a NucleoSpin Blood QuickPure kit (Macherey-Nagel) following the manufacturer’s recommendations. The serological status of animals for SRLV infection was determined using the commercially available ID Screen MVV/CAEV Indirect Screening Test (IDvet, France). For molecular characterization of proviral DNA, the V4/V5 (608 bp) fragment of the *env* gene and a 625-bp fragment of the *gag* gene encoding the CA protein were amplified by nested PCR. The primers Ptat/Penv and 567/564 were used in the first and second round of PCR, respectively, for amplification of the V4/V5 fragment [12, 20]. For amplification of the *gag* gene fragment, the primer pairs GAGf1 and P15 were used in the first round, and CAGAG5 and CAGAG3 were used in the second round [12, 21]. PCR products were analyzed by electrophoresis on a 2% agarose gel containing ethidium bromide (1 µg/ml) in 1x TAE buffer. DNA samples were purified from agarose gels using a NucleoSpin Extract II Kit (Marcherey-Nagel) and sequenced on a 3730x1 DNA Analyzer (Applied Biosystems) using a Big Dye Terminator v3.1 Cycle Sequencing Kit. To examine the genetic variability of analysed SRLV strains, we performed direct sequencing of the amplified products, which, despite misincorporations, yielded a consensus sequence for each strain that was identical to that of the starting template. Sequence data were analysed using the Geneious alignment module within Geneious Pro 5.3 software (Biomatters Ltd). Phylogenetic analysis was performed using the Geneious tree-builder tool, and phylogenetic trees were constructed using Bayesian inference with the GTR substitution model. Pairwise genetic distances were calculated using the MEGA 6 software application [22] according to the p-distance substitution model with default settings, except for ignoring all sites with gaps. The sequences obtained in this study were deposited in the GenBank database with accession numbers MH790874- MH790887.

Serological and molecular screening of animals from mixed flocks showed substantial differences in the proportion of infected animals with respect to the animal species and the diagnostic test used. Out of 68 sheep serum samples, 22 were positive in ELISA, while no positive serological results were obtained with samples from goats. Consistent with this, none of the samples from goats gave a positive amplification signal in PCR. Table 1 shows the distribution of PCR test results for specimens collected from 22 seropositive sheep. A 625-bp fragment of the *gag* gene was successfully amplified and sequenced from 14 samples. We failed to amplify samples 6918, 1759, and 7048 from flock 1, samples 7060 and 7497 from flock 2, sample 1762 from flock 3, and samples 3371 and 1423 from flock 4. Amplification of the V4/V5 fragment gave a specific product of the expected size in one sample only (sample 6922).

**Table 1:** Origin and characteristics of SRLV strains found in sheep

Flock	Animal no.	Commercial ELISA	PCR (*gag*)	PCR (*env*)	Subtype
1	6922	+	+	+	A13
	8063	+	+	-	A13
	9179	+	+	-	A13
	6918	+	-	-	-
	1759	+	-	-	-
	7041	+	+	-	A18
	7048	+	-	-	-
2	0090	+	+	-	A18
	7016	+	-	-	-
	7010	+	+	-	A18
	7020	+	+	-	A18
	7497	+	-	-	-
3	1762	+	-	-	-
4	6981	+	+	-	A13
	9155	+	+	-	A13
	1406	+	+	-	A13
	3371	+	-	-	-
	4742	+	+	-	A18
	1304	+	+	-	A13
	6969	+	+	-	-
	1423	+	-	-	-
	1911	+	+	-	A13

All 14 *gag* sequences were aligned with reference sequences available in the GenBank database, representing the SRLV genotypes described to date. As many geographically diverse strains as possible were included in this analysis; however, only sequences of length matching the data generated in this study were examined. An unrooted phylogenetic tree was constructed using the Bayesian method as shown in Fig. 1. All of the tested samples belonged to SRLV group A. Nine sequences clustered with strain 0016 from subtype A13, which was supported by a high posterior probability of 0.9. The mean nucleotide sequence divergence of these nine isolates and strain 0016 was 5.0% (Table 2). Five sequences formed a new cluster within group A, which was tentatively named A18 (strains 4742, 7020, 0090, 7010 and 7041). The mean nucleotide sequence divergence between isolates in this new cluster and other subtypes of group A (A1-A5, A8-A9, A11-A13, A16-A17) varied from 12.7% to 18.9% (Table 2). Additionally, the existence of the new subtype was supported by high posterior probability (0.92). Thus, we propose to distinguish A18 as a new subtype within group A. Circulation of more than one subtype, A13 and A18, was found in two flocks (1 and 4), while all isolates from flock 2 clustered together in the new subtype A18.

**Fig. 1:**
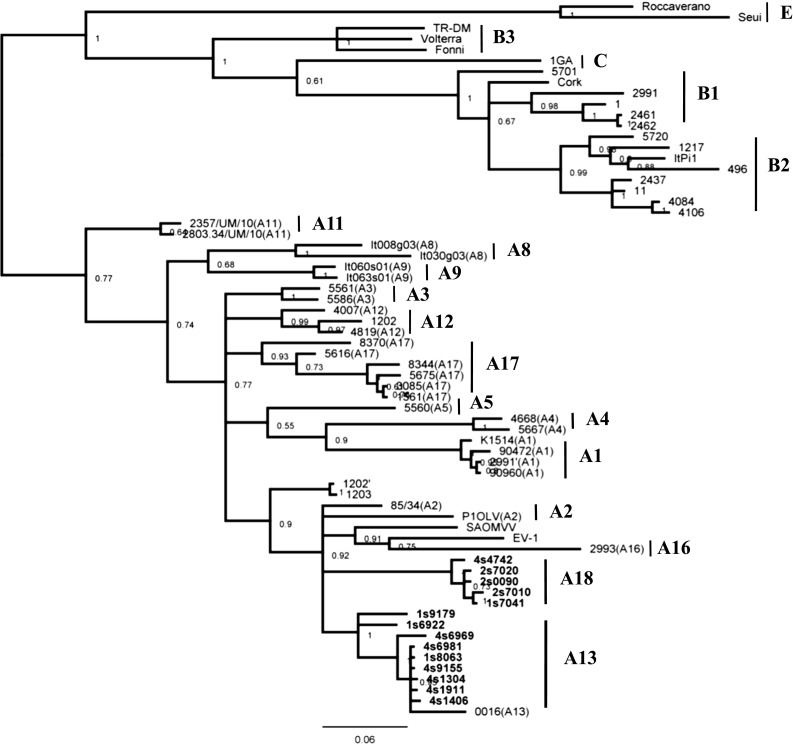
Bayesian phylogenetic tree based on the CA fragment. Sequences from this study are shown in bold, and their names are preceded by the flock number and animal species (s-sheep)

**Table 2:** Mean nucleotide distance in the CA fragment of the *gag* gene between genotypes A and B of SRLV

	B1	B2	B3	A1	A2	A3	A4	A5	A8	A9	A11	A12	A13	A16	A17
B1	-	-	-	-	-	-	-	-	-	-	-	-	-	-	-
B2	12.0	-	-	-	-	-	-	-	-	-	-	-	-	-	-
B3	15.0	14.0	-	-	-	-	-	-	-		-	-	-	-	-
A1	26.2	23.4	23.3	-	-	-	-	-	-	-	-	-	-	-	-
A2	23.9	21.9	22.2	18.1	-	-	-	-	-	-	-	-	-	-	-
A3	22.1	21.4	20.9	16.1	15.7	-	-	-	-	-	-	-	-	-	-
A4	23.4	21.8	23.2	16.8	16.6	16.5	-	-	-	-	-	-	-	-	-
A5	22.8	21.1	22.9	15.6	15.8	12.5	14.3	-	-	-	-	-	-	-	-
A8	24.0	23.3	22.4	17.5	18.9	16.3	17.4	18.1	-	-	-	-	-	-	-
A9	23.1	21.9	20.7	16.6	17.4	13.7	16.1	14.3	15.1	-	-	-	-	-	-
A11	21.8	22.5	21.5	16.6	17.7	15.5	18.8	16.2	16.9	14.9	-	-	-	-	-
A12	23.0	22.8	22.4	18.0	15.0	12.4	17.2	13.6	17.0	16.4	15.2	-	-	-	-
A13	22.5	19.9	21.0	15.7	12.5	13.0	15.4	14.9	17.0	16.0	14.5	13.4	-	-	-
A16	22.9	22.4	24.1	19.0	16.7	17.8	16.0	16.1	18.3	19.0	18.8	17.8	16.0	-	-
A17	22.1	21.2	21.6	15.3	14.6	11.0	15.0	12.6	16.5	12.8	16.4	12.7	14.3	16.4	-
**A18**	**23.9**	**22.4**	**21.7**	**18.9**	**14.3**	**15.2**	**17.4**	**17.2**	**18.8**	**17.2**	**17.6**	**14.7**	**12,7**	**16.8**	**14.7**

In order to analyse the sequence conservation of immunodominant regions of the Gag protein, the deduced amino acid (aa) sequences of *gag* fragments of newly identified strains were compared with aa sequences of reference strains and strains that are representative of subtypes previously found in Poland. Out of three immunodominant epitopes identified in the Gag protein [23], two were accessible for this study (Fig. 2).The immunodominant epitope 2, situated at the N-terminal end of the CA protein, was highly conserved within all strains representing the A subtype, except strain 6969 from the A13 subtype, which differed by three aa changes, glutamine (E) to asparagine (D), alanine (A) to glycine (G), and glutamine (Q) to histidine (H). Immunodominant epitope 3, from the C-terminal end of the CA protein, was also well conserved among A-subtype strains, except one strain (4742) with a single aa substitution, glutamine (Q) to histidine (H).

**Fig. 2:**
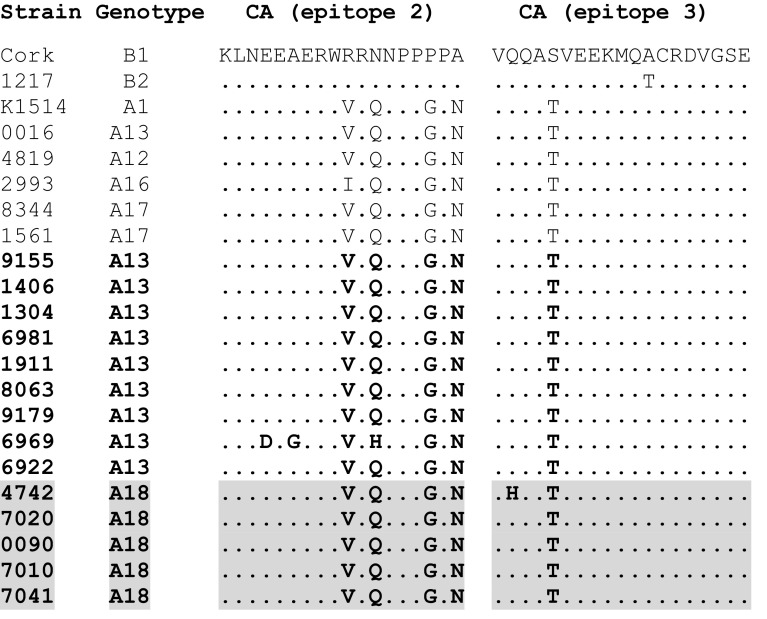
Sequence homology of Gag immunodominant epitopes between strains analyzed in this study (marked in bold) with reference strains from genotype A1 (K1514), B1 (Cork), B2 (1217), A13 (0016), A12 (4819), A16 (2993) and A17 (8344, 1561). The sequences of the new subtype A18 strains are shaded

A previous report showed that the SRLV population diversity in Poland is rather complex, which probably reflects the absence of any SRLV eradication program linked to the culling of infected individuals. Phylogenetic analysis revealed the circulation of subtypes A1, A12, and B2 in both sheep and goats, subtypes B1, A16 and A17 in goats, and subtype A13 only in sheep [11, 12]. We also observed that circulation of subtypes B1, B2 and A1 was widespread throughout the country, while some particular subtypes may be geographically restricted to specific regions. Subtypes A17, A16, A13 and A12 were detected only in the Wielkopolska, Podkarpackie, Malopolska and Podlaskie region, respectively. The present study provides a more detailed picture of SRLV strains circulating in the Malopolska region, which is characterized by the highest seroprevalence of SRLV in the country and frequent breeding of sheep and goats in mixed flocks.

Surprisingly, in the present study, only sheep from mixed flocks were positive for SRLV infection, while samples from goats were negative by ELISA and PCR. Moreover, only strains from subtype A13, previously described in sheep in Poland, and the newly proposed subtype A18 were detected in sheep. Therefore, no evidence of cross-species transmission was obtained in this study. Our previous study [17] showed that sheep in this region had the highest SRLV seroprevalence in Poland, but no data on the seroprevalence in goats were available. Taking into account that the overall prevalence of SRLV in goats in Poland is high [16], we presumed that we would be able to find seropositive goats. We were very surprised that all of the goats tested were seronegative. Furthermore, we did not expect that we would find only MVV-like sequences in sheep, because CAEV-like viruses have been identified quite frequently in sheep in Poland. We can rule out false negative results in the nested PCR when genomic DNA was amplified from goats, since the primers used in this study were able to amplify both CAEV- and MVV-like sequences.

Nine out of fourteen SRLV sequences obtained (65%) belonged to subtype A13, which, to date, is represented exclusively by only one strain (0016), isolated 6 years ago from sheep in the Malopolska region [12]. Therefore, subtype A13 may be considered as predominant in this region. The present study also revealed the existence of a new subtype circulating in this area. The sequences of strains 4742, 7020, 0090, 7010 and 7041 were found to belong group A, but they were segregated into a separate cluster that was quite distinct from the known subtypes and supported by a high posterior probability. The mean pairwise genetic distances of *gag* sequences were above 15% nucleotide sequence divergence when compared to all other subtypes within group A, which is a criterion for establishing a new subtype [7]. Also, unsuccessful amplification of *env* fragments in 13 out of 14 samples may suggest high sequence divergence of these strains from sequences of well-known genotypes. Therefore, we propose to tentatively name the new subtype “A18”.

It was notable that this new subtype was present in almost all of the flocks tested, like subtype A13. This suggests broad dissemination of both subtypes in the Malopolska region. We can assume that these flocks, in some cases situated several dozen kilometers apart, were epidemiologically linked to each other, likely by trade practices or by common traditional grazing of sheep on mountain pastures. It is common knowledge that the introduction of an infected sheep into a flock represents an important risk factor for the dissemination of SRLV infections [24]. Also, the presence of both subtypes in sheep from the Malopolska region raises questions about the origin of these viruses. Maedi visna in sheep was probably introduced to Poland through the movement of animals together with German settlers during World War II [25] and then, along with the growing sheep population and sheep industry in the 1970s, the disease became prevalent in the entire country. Despite the fact that SRLV strains currently identified within subtypes A1, A12, A13, A16, A17, and A18 showed nucleotide pairwise distances ranging from 12.7% to 19.0%, we can suppose that these viruses could have been the original ancestors and that the diversification into predominant subtypes was possibly due to the effect of a “founder virus” and genetic drift or selection pressure [10].

In summary, the results of this work extend the current knowledge of the distribution of SRLV subtypes in sheep and goats in Poland and provide further evidence of the genetic variability of SRLV. This study provides new data on the distribution of different SRLV subtypes and the existence of a new subtype, which may be of practical value for both epidemiological studies supporting local eradication programs and evolutionary studies of SRLV. Furthermore, the characterization of sequences encoding antigenic determinants highlights the potential effects of genetic variability on serological detection. Our results show that SRLV strains from the Malopolska region maintain a high degree of conservation within immunodominant epitopes 2 and 3 of the Gag protein. Conservation of these epitopes is crucial for maintaining appropriate cross-reactivity in serological tests, which is of key importance for eradication programs. On the other hand, sequencing of a longer DNA fragment will be necessary to investigate the occurrence and significance of genetic diversity and its possible effect on serological tests. In our study, using multi-epitope recombinant antigens derived from different subtypes of SRLV, we noted that the serological status of field serum samples strongly depends on the type of antigen that was used. We also found evidence (to be presented in a future publication) that standard commercial ELISAs may fail to detect infection in a proportion of the animals, influencing the control strategy applied in the field. Therefore, we emphasize the need to perform extensive study on the association between antigenic variability of SRLV and the performance of diagnostic tests. In particular, seronegative animals should be investigated for infections by new genotypes that are not detected by traditional serological tests. Also, further isolation and characterization of viruses belonging to subtype A13 and the new subtype A18 may shed light on their biological properties.
